# Effects of Defects on Bond Behavior of Fiber Reinforced Cementitious Matrix Materials

**DOI:** 10.3390/ma13010164

**Published:** 2020-01-01

**Authors:** Antonio Bilotta, Gian Piero Lignola

**Affiliations:** DiSt Department of Structures for Engineering and Architecture, University of Napoli Federico II, Via Claudio, 21, 80125 Naples, Italy; antonio.bilotta@unina.it

**Keywords:** bond test, FRCM, cementitious matrix, fiber, strengthening system, bond behavior

## Abstract

High-strength fibers embedded in inorganic matrix i.e., Fiber Reinforced Cementitious Mortar materials (FRCM) are commonly used as strengthening technique for existing masonry structures, due to the low sensitivity to debonding phenomena between substrate and matrix. Nevertheless, the use of lime or cement-based matrix instead of epoxy adhesive implies that attention has to be paid to the bond behavior between the fibers and the matrix, since sliding phenomena and cohesive failures in the mortar matrix can occur. The paper aims to investigate the effect of the mechanical properties of fiber and matrix on the FRCM efficiency, and potential geometrical defects, typical of real applications. The aim is to analyze the mechanical behavior of the FRCM system by simulating hypothetical bond tests, as they are usually performed in laboratories. The bond test has a significant role, as it is used for the qualification of the material, providing sometimes very scattered results. Hence, it is particularly important and greatly discussed in the scientific community and among manufactures and practitioners. The purpose is to understand where this variability could derive from and possibly how to contain it, to improve the characterization of FRCM systems. A mechanical model has been proposed to simulate the usual bond test to focus and stress the way in which each fiber slips out of the matrix as the load increases; and this has been recognized as the main reason for scattered results in bond tests. The model was then applied to the typical cases of PBO-FRCM and Glass-FRCM, hence considering different ratios for the fiber and matrix properties.

## 1. Introduction

In recent years, Civil Engineering experienced a rapid development of new technologies aimed at consolidating the existing buildings. A strong impulse was determined by the need to repair structures after recent seismic events and, above all, to guarantee an adequate level of seismic safety with respect to future events, hence, to strengthen existing structures. Techniques based on the use of fiber-reinforced composite materials represent an important technological innovation that is increasingly used for reinforced concrete and masonry structures. The use of Fiber Reinforced Polymer (FRP) composites, made of fibers immersed in polymeric matrices—usually epoxy resins— has become a common practice in the last decade; it guarantees a series of advantages, including high strength-to-weight ratios (useful to reduce the increase of overall structural mass) and high resistance to corrosion. Nevertheless, problems related to low resistance to fire and high temperatures, the relatively high cost of resins, the impossibility of applying FRP on wet substrates or at low temperature, the lack of vapor permeability, and the mechanical incompatibility of epoxy resins with poor materials such as the masonry, pushed the industry to identify alternative matrices. 

To date, Fiber Reinforced Cementitious Matrix/Mortar (FRCM), also known as TRC (Textile Reinforced Concrete), TRM (Textile Reinforced Mortar), FRM (Fabric Reinforced Mortar) or IMG (Inorganic Matrix-Grid) Composites, are added to the classic FRP composites. These composites are obtained by applying one or more reinforcing grids into an inorganic matrix (or mortar) that can be cement-based, lime-based or geopolymeric, obtaining a mechanical behavior quite different from the traditional FRP [1,2,3,4,5,6]. As far as fibers are concerned, they can be made of glass, carbon, aramid, basalt, PBO (Poliparafenilenbenzobisoxazolo) or steel.

FRCM are directly made onsite and bonded to the support to be strengthened. The mechanical behavior of a composite system depends on the characteristics of the individual constituent materials and, above all, on their interactions, strongly related to the adherence between the materials at their interface.

The interaction between fiber and matrix is of fundamental importance for the performance of the entire system since it determines the transfer of the stresses from the fibers to the surrounding matrix, and consequently to the support. The most critical part of these types of systems is surely the fiber-matrix interface rather than the matrix-support interface, as it is generally the case for the FRP reinforcement.

Although these materials are very promising for increasing the mechanical properties of existing structures, to date, the understanding of the mechanical properties of FRCMs, the experimental procedures for their mechanical characterization, and the modeling and the formulation of appropriate design criteria, are problems still discussed in the field. The main difficulty is to manage their heterogeneous nature, which produces a strongly non-linear mechanical behavior, making the interaction with the substrate, over which they are applied, even more complex [1,7,8,9,10,11,12,13,14,15].

**Figure 1:**
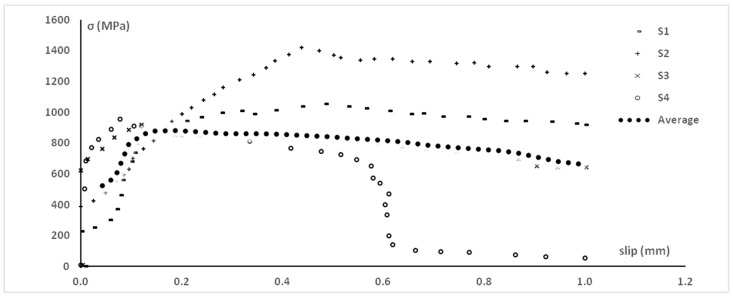
One of the sets from a laboratory of stress-slip responses of the PBO-FRCM (Fiber Reinforced Cementitious Mortar) system tested in the round robin test (adapted from [16]).

Numerous theoretical and experimental studies have been conducted to characterize the mechanical behavior of FRCMs by investigating the local effects of adherence. [16] studied the performances of two types of FRCM characterized by different fibers made of PBO, [17] showed the results of tensile tests performed on FRCM with basalt fibers, [18] with glass fibers, [19] with PBO and aramid fibers, and [20] with steel fibers, all of them in a round robin test. Large variability was found not only among different laboratories, but in each laboratory, too (e.g., Figure 1 shows a series of bond test results on PBO-FRCM systems), remarking the need to understand where this variability could derive from and possibly how to contain it, to improve the characterization of FRCM systems. It could be ascribed mainly to the FRCM system rather than on setup, as such variability was not found in the case of FRPs [21,22].

**Figure 2:**
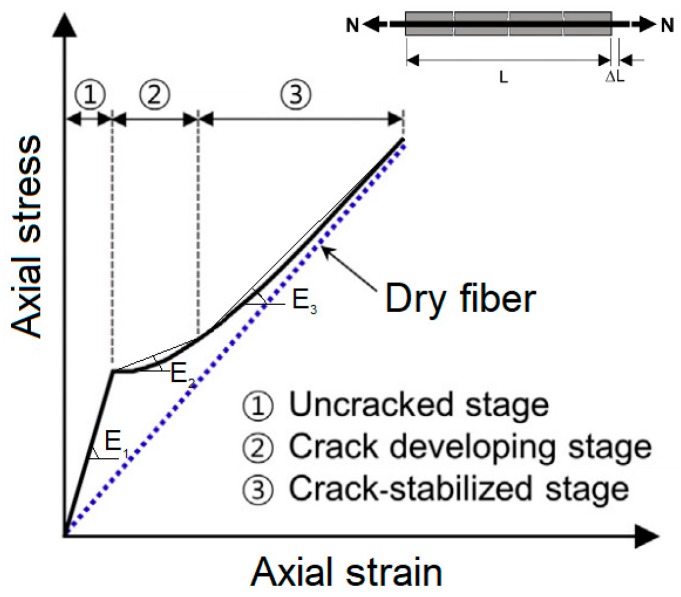
Tensile *σ*-*ε* relationship of a FRCM specimen.

Based on these results, [23] proposes that the tensile response of FRCMs can be divided into three phases as illustrated in Figure 2:

- Phase 1 (uncracked stage): The matrix is not yet cracked and the mechanical tensile behavior is governed by the mechanical properties of the matrix;
- Phase 2 (crack developing stage): The tensile strength of the matrix is exceeded, hence cracking begins to take place and the load is gradually transferred to the fibers;
- Phase 3 (crack-stabilized stage): The load is almost completely transferred to the fibers and the mechanical tensile behavior is governed by the mechanical properties of the fibers (i.e., *E*_3_ ≅ *E_f_*).

Hence, the tensile response is considered to involve three branches with almost constant slopes (*E*_1_, *E*_2_ and *E*_3_), howsoever the third phase could be missing in some cases, depending on combinations of fibers and matrix properties.

Nevertheless, this relationship is not sufficient to characterize the mechanical behavior of a FRCM system [24], since for the strengthening of structural elements multiple failure modes may occur because of substrate-to-FRCM interaction, as listed below:

- Debonding with cohesive failure of the support of the reinforcement;
- Debonding at the matrix interface-support;
- Debonding at the matrix-fiber interface;
- Slip of the fiber inside the matrix;
- Slip of the fiber and cracking of the outer layer of mortar;
- Tensile failure of the fiber.

For these reasons, in the following, a mechanical model will be proposed to simulate the bond process during testing to examine the way in which the fiber slips out of the matrix as the load increases. The model was then applied to the case of PBO-FRCM and Glass-FRCM.

## 2. Bond Test Simulation

The typical bond test is the “single-lap shear test”, where the FRCM system is applied on one side of the masonry block. In particular, the paper focuses on the simulation of a bond test performed on a single bundle of fibers immersed inside a mortar matrix, in adhesion to an infinitely rigid support, as the substrate can be assumed compared to the FRCM system (as seen with Digital Image Correlation, DIC, technique also by [25]). In order to carry out the analyses with the numerical algorithm, it is necessary to define the geometrical and mechanical properties of the fiber and matrix, i.e., the (*σ*;*ε*) constitutive relationship and the bond properties at the fiber-matrix and matrix-support interface, i.e., the (*τ*;*s*) relationship. The symbols are listed in Table 1.

### 2.1. Material Constitutive Laws

The matrix constitutive relationship in tension (*σ*;*ε*) is assumed linear elastic up to failure at a stress level, *f_t_*.

(1) $$\sigma_{m}=E_{m}\cdot \varepsilon_{m}<f_{t}$$

The same relationship is assumed for the fiber in tension. To simulate a not homogeneously stretched bundle, as potentially could occur during the installation, a non-linear constitutive relationship can be used. The “defect” is simulated, considering that the bundle deforms with a variable stiffness, lower than *E_f_*, until it is perfectly stretched to be loaded with its proper relationship. This is modeled by determining a strain or stress at the perfect straightening of the bundle.

In the constitutive relationship, the defect is expressed through the strain *ε_def_*, which corresponds to the transition stress *σ_def_* from the non-linear behavior, in the straightening phase, to the linear behavior. In particular, the first branch of the *σ-ε* relationship with defect is assumed parabolic

(2) $$\sigma_{f}=a\cdot \varepsilon_{f}{}^{2}$$

with a horizontal tangent at the beginning, namely at point (*ε* = 0 and *σ* = 0) and the slope *E_f_* at the end, namely at point (*ε_def_*, *σ_def_*).

Being useful to express the strain as a function of the stress, in strain compatibility problems, the function can be manipulated as:

(3) $$\varepsilon_{f}=\sqrt{\frac{\sigma_{f}}{a}}$$

and

(4) $$\frac{d\sigma_{f}}{d\varepsilon_{f}}=2a\cdot \varepsilon_{f}$$

Assumed that, at point (*ε_def_*, *σ_def_*), the slope of the relationship is *E_f_*, it is:

(5) $$\frac{d\sigma_{f}}{d\varepsilon_{f}}=2a\cdot \varepsilon_{f,def}=E_{f}$$

and thus derive:

(6) $$a=\frac{E_{f}}{2\cdot \varepsilon_{f,def}}$$

By substituting the Equation (6) into Equation (3):

(7) $$\varepsilon_{f}=\sqrt{\frac{\sigma_{f}\cdot 2\cdot \varepsilon_{f,def}}{E_{f}}}$$

Hence it is clear that:

(8) $$\varepsilon_{f,def}=\frac{2\cdot \sigma_{f,def}}{E_{f}}$$

Therefore, for each *E_f_*, the magnitude of the defect is completely defined by one of the two: *ε_f_*_,*def*_ or *σ_f_*_,*def*_.

Finally, the elastic branch for *ε_f_* > *ε_f_*_,*def*_ up to failure is defined through the following relationship:

(9) $$\varepsilon_{f}=\varepsilon_{f,def}+\frac{\sigma_{f}-\sigma_{f,def}}{E_{f}}=\frac{\sigma_{f,def}+\sigma_{f}}{E_{f}}$$

Obviously, this relationship $\varepsilon_{f}=f\left(\sigma_{f}\right)$ is completely linear elastic if *ε_f_*_,*def*_ = 0 or equivalently if *σ_f_*_,*def*_ = 0.

**Figure 3:**
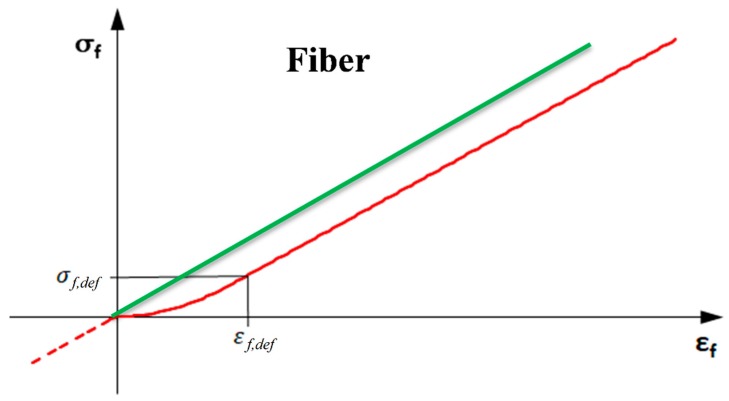
*σ*-*ε* relationship without and with defect.

Note that the constitutive relationship is defined also at negative stress and strain (see Figure 3) to prevent that the numerical algorithm fails for oscillations at low stresses close to zero, howsoever it is not admitted that the fibers are in compression.

Therefore, the constitutive relationship in Figure 3 is analytically expressed by Equation (10):

(10) $$\varepsilon_{f}=\left\{\begin{array}{ccc} \frac{\sigma_{f}}{E_{f}} & if & \varepsilon_{f,def}=\sigma_{f,def}=0\\ \frac{\sigma_{f}}{E_{f}} & if & \sigma_{f}\leq 0\\ \sqrt{\frac{\sigma_{f}\cdot 2\cdot \varepsilon_{f,def}}{E_{f}}} & if & 0\leq \sigma_{f}\leq \sigma_{f,def}\\ \varepsilon_{f,def}+\frac{\sigma_{f}-\sigma_{f,def}}{E_{f}} & if & \sigma_{f}>\sigma_{f,def} \end{array}\right\}$$

### 2.2. Interface Bond Relationships

**Figure 4:**
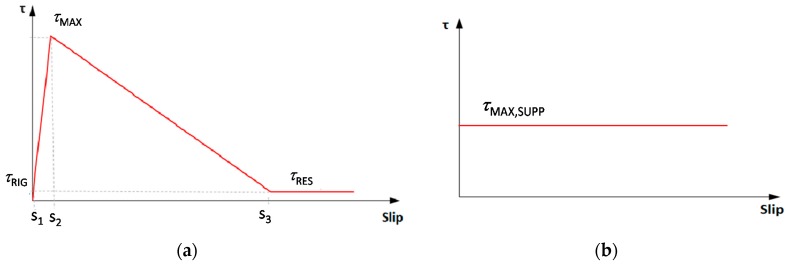
*τ*-*s* relationship (**a**) fiber-matrix interface and (**b**) matrix-support interface.

Figure 4a shows the bond behavior by means of the relationship between the tangential stress τ, which arises at the fiber-matrix interface, and the slip *s*, defined as the difference between the displacement of the fiber and that of the matrix. Based on several experimental studies, this shape can be considered suitable for different types of fiber and matrix, if the values $\tau_{RIG}$, $\tau_{MAX}$, $\tau_{RES}$ and the corresponding slips are properly assumed for different fibers (e.g., PBO, steel, glass, etc.), despite the differences in chemical-physical properties at the interfaces. Therefore, the bond relationship is expressed as:

(11) $$\tau \left(s\right)=\{\begin{array}{ccc} 0 & if & s<0\\ \frac{\tau_{RIG}}{s_{1}}\cdot s & if & s<s_{1}\\ \tau_{RIG}+\frac{\tau_{MAX}-\tau_{RIG}}{\begin{array}{c} s_{2}-s_{1} \end{array}}\cdot \left(s-s_{1}\right) & if & s<s_{2}\\ \tau_{MAX}-\frac{\tau_{MAX}-\tau_{RES}}{\begin{array}{c} s_{3}-s_{2} \end{array}} & if & s<s_{3}\\ \tau_{RES} & if & s>s_{3} \end{array}$$

The bond relationship between the FRCM matrix and its substrate correlates the tangential stresses—which arise at the interface—to the slips between matrix and substrate. In the simplified assumptions of infinitely rigid support that has no displacement, when the FRCM system is in tension, the slip coincides exactly with the displacement of the FRCM only. Since the analytical model is limited to considering the slip between fiber and matrix, i.e., the difference of their displacements, the value of each of them can be calculated without this absolute displacement. For simplicity, we assume that there is a perfectly plastic behavior between the matrix and the support, due to a cohesive bond mechanism with maximum tangential stress, $\tau_{MAX,SUPP}$, constant for any slip value (see Figure 4b). Generally, in practical applications the shear bond demand to the support is lower than available shear bond capacity; conversely, the shear bond capacity of fibers to matrix is usually lower in the case of FRCM compared to the case of FRP.

## 3. Single Bundle Algorithm

**Figure 5:**
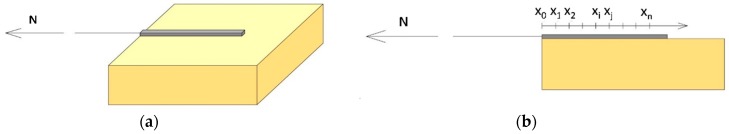
Single bundle bond test (**a**) 3D view and (**b**) geometry of the model.

Figure 5 shows the geometry of the system, to set the algorithm. Subscripts i and j refer to two sections located at a distance ∆*x*. The values at the loaded end section by the force N are marked with the subscript 0, while those at the final section are marked with the subscript n. The symbols are listed in Table 2.

The anchorage length, *L*, is the length required to allow the transfer of the force *N* acting in the bundle to the surrounding matrix, by means of bond stresses. The first purpose of the algorithm is to identify the section *x_n_* (see Figure 5) where the anchoring of the bundle is finally guaranteed.

### 3.1. General Problem

For each section x, axial strain in the fiber and in the matrix and slip can be defined as:

(12) $$s=u_{f}-u_{m}$$

(13) $$\varepsilon_{f}=\frac{du_{f}}{dx}$$

(14) $$\varepsilon_{m}=\frac{{du}_{m}}{dx}$$

The derivative of Equation (12) with respect to *x*, by considering the Equations (13) and (14) provides:

(15) $$\dot{s}=\frac{ds}{dx}=\frac{du_{f}}{dx}-\frac{du_{m}}{dx}=\varepsilon_{f}-\varepsilon_{m}$$

The perfect anchorage of the bundle in the matrix is finally guaranteed when the slip and its derivative are zero:

(16) s=0

(17) $$\dot{s}=0$$

that means that the displacement of the fiber and the matrix and the strain are equal to each other.

Without any bond to the support, (i.e., in the case of tensile test), the longitudinal equilibrium at the lateral boundary of an elementary section of bundles of length ∆*x*, provides the following equation:

(18) $$\pi \cdot \Phi \cdot \tau \left(s_{m}\right)\cdot \Delta x=\pi \cdot \frac{\Phi^{2}}{4}\cdot d\sigma_{f}$$

Hence:

(19) $$\tau \left(s_{m}\right)=\frac{\Phi }{4}\cdot \frac{d\sigma_{f}}{\Delta x}$$

Equation (19) indicates that, due to the tangential bond stresses, the tension in the bundle is not constant. Obviously, the stress reduces by moving away from the loaded end section, or from a potentially cracked section, where traction is applied to the bundle only. Moreover, for longitudinal equilibrium of the entire section, assuming the conservation of plain sections, the following equation can be written:

(20) $$A_{f\cdot }\sigma_{f}+\int_{A_{tm}}\sigma_{m}\cdot dA=N$$

Despite the reduction of normal stress that occurs locally in the bundle, this corresponds to an increase of normal stress in the matrix. In the case of bond tests, the contextual transfer between fiber and matrix and between matrix and substrate should be taken into account. Therefore, as already mentioned in the previous section, the bond between the matrix and the support shall be considered because the matrix is not loaded at the same level as the bundle is unloaded.

### 3.2. Numerical Procedure

The problem stated by Equations (19) and (20) can be solved through discretization by means of finite differences by arbitrarily assigning a trial value of the slip $s_{i}=s_{0}$ at the end section, that is preferably the origin of the reference *x*-axis. The procedure is iterated with a “trial and error” method, until the boundary condition in the other section is satisfied.

In particular, the following relationship can be written at the loaded end section (i.e., where the tensile force is applied only to the bundle):

(21) $$\sigma_{f_{i}}=\sigma_{f_{0}}=\frac{N}{A_{f}}$$

(22) $$\sigma_{m_{i}}=\sigma_{m_{0}}=0$$

(23) $$\varepsilon_{f_{i}}=\varepsilon_{f_{0}}=\frac{\sigma_{f_{0}}}{E_{f}}$$

(24) $$\varepsilon_{m_{i}}=\varepsilon_{m_{0}}=0$$

Assuming a distance, ∆*x*, between sections *x_i_* and *x_j_*, first trial slip $s_{i}=s_{0}$ and $\Delta s=0.01\cdot s_{0}$ are assumed to estimate:

(25) $${\left(\frac{\Delta s}{\Delta x}\right)}_{TRIAL-i,j}=\frac{s_{i}-s_{j}}{\Delta x}$$

The limit for that ratio as Δ*x* approaches zero is the first derivative of the slip, $\dot{s}$. The average slip can be calculated by means of the following relationship:

(26) $$s_{m-i,j}=\frac{s_{i}+s_{j}}{2}$$

The bond relationship in Equation (11) provides the corresponding average shear stress, $\tau \left(s_{m-i,j}\right)$.

**Figure 6:**
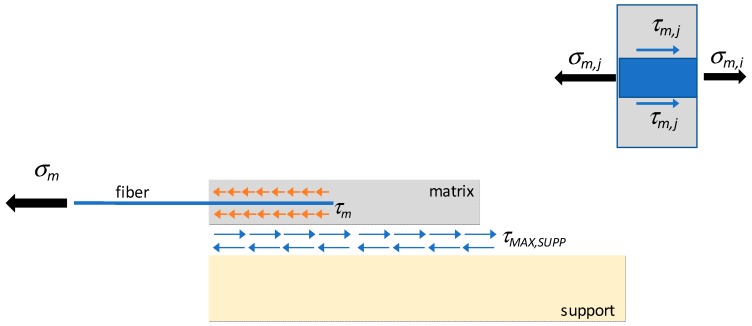
Shear transfer at fiber-matrix (*τ_m_*) and matrix-support ($\tau_{MAX,SUPP}$) interface.

Since the matrix is loaded by the fiber and, at the same time, it is loading the substrate (as far as is possible depending on the value of maximum tangential stress $\tau_{MAX,SUPP}$), the stress in the matrix and the shear stress at the matrix-support interface at *x_j_* are obtained through translation equilibrium (see Figure 6):

(27) $$\sigma_{m_{j}}=MAX\left(\sigma_{m_{i}}+\tau \left(s_{m-i,j}\right)\cdot \Delta x\cdot \frac{\pi \cdot \Phi }{A_{tm}}-\tau_{MAX,SUPP}\cdot \frac{\Delta x\cdot b}{A_{tm}};0\right)$$

(28) $${\bar{\tau }}_{j}=MIN\left(\frac{\sigma_{m_{i}\cdot \;}A_{tm}}{\Delta x\cdot b}+\frac{\tau \left(s_{m-i,j}\right)\cdot \pi \cdot \Phi }{b};\tau_{MAX,SUPP}\right)$$

If the value of $\tau_{MAX,SUPP}$ at the matrix-support interface is higher than the shear capacity required to transfer the stresses to the substrate, the tension of the matrix is transferred entirely to the support. Otherwise, the first terms of (27) and (28) provide the tensile stress in the matrix and shear stress at the matrix-support, respectively, by taking into account the simultaneous presence of the two bond transfers.

Therefore the load in the FRCM at *x_j_* is:

(29) $$N_{oj}=N_{oi}-{\bar{\tau }}_{j}\cdot b\cdot \Delta x$$

Note that at the loaded end section $N_{oi}=N$.

Finally, the axial stress in the fiber at *x_j_* is:

(30) $$\sigma_{fj}=\frac{N_{oj}-\sigma_{m_{j}}\cdot A_{tm}}{A_{f}}$$

and the matrix strain and fiber stain can be obtained by means of Equations (1) and (10).

With the average strain, through the Equation (15), the first derivative of the slip can be calculated:

(31) $${\dot{s}}_{i,j}={\left(\frac{ds}{dx}\right)}_{i,j}=\frac{\varepsilon_{f}{}_{i}+\varepsilon_{f}{}_{j}}{2}-\frac{\varepsilon_{m}{}_{i}+\varepsilon_{m}{}_{j}}{2}$$

The procedure, iterative by varying s_0_, ends when the condition (32) is satisfied:

(32) $${\left(\frac{ds}{dx}\right)}_{i,j}-{\left(\frac{\Delta s}{\Delta x}\right)}_{TRIAL-i,j}=0$$

The values $\sigma_{m_{j}},\sigma_{fj,}\varepsilon_{m_{j}},\varepsilon_{f_{j}}$ calculated at *x_j_* are used to start the iteration between the section *x_i_*_+1_ and *x_j_*_+1_ and so on. The algorithm is iterated up to the section at *x_n_* where condition (16) and (17) are satisfied. In the programming instructions, the following condition has been used:

(33) $$s_{j}>0\;\;\;\;\;\;\wedge \;\;\;\;\;\;\dot{s_{j}}>0$$

This position is necessary because if the slip becomes negative, the matrix has greater displacement than the bundle, and if the derivative of the slip becomes negative, the matrix has strain greater than that of the bundle; both situations have no physical sense.

Obviously, for numerical reasons, the condition *s_n_* = 0 is implemented numerically by means of a threshold value (i.e., $s_{n,TARGET}=\frac{1}{100}\cdot s_{2}$ where *s*_2_ is the maximum slip value in the bond relationship). 

If the iteration led to *s_n_* < 0, the value of s_0_ was too low. If *s_n_* > *s_n,TARGET_*, the value of *s*_0_ was too high.

The distance between *x*_0_ and *x_n_* is the length necessary to guarantee the perfect bond between the bundle and the matrix (otherwise other failure modes are attained). Hence, a residual axial stress in the bundle can be transferred to the matrix through a constant stress distribution at the interface (e.g., Brice model, [26]) requiring a length $L_{b}=\frac{\Phi }{4}\cdot \frac{\sigma_{n}}{\tau_{b}}$ where *τ_b_* = *τ_RIG_*. Finally, the total anchoring length is *L* = *L’ + L_b_*.

### 3.3. Example of Some Numerical Results

**Figure 7:**
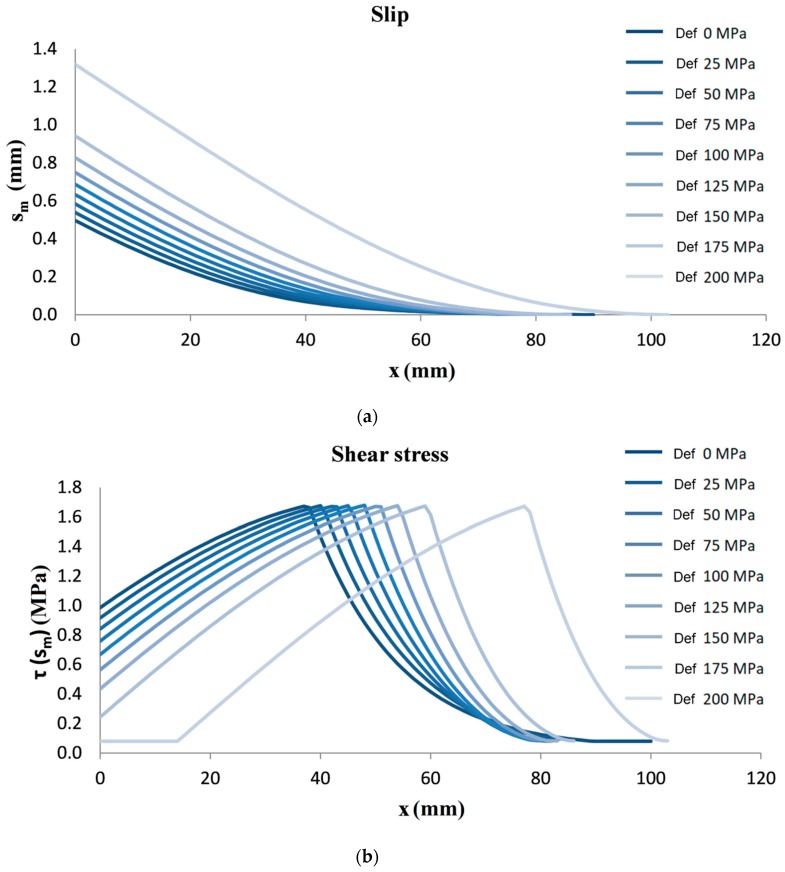

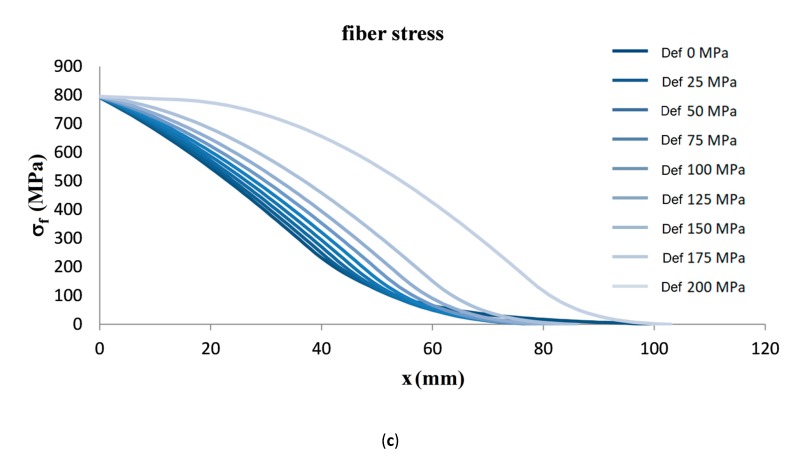
Results of numerical simulations: (**a**) Average slip, $s_{m}\left(x\right)$, (**b**) average shear stress, τ(x), (**c**) average stress in the fiber, $\sigma_{f}\left(x\right)$, along the G-FRCM.

Using the proposed algorithm, it is possible to obtain average slip, $s_{m}\left(x\right)$, average shear stress, τ(x), average stress in the fiber, $\sigma_{f}\left(x\right)$, and average stress in the matrix, $\sigma_{m}\left(x\right)$, versus the abscissa *x*, for different defect levels. Figure 7 shows that slip increases with the defects, and the shear stress profiles are translated along the FRCM because at higher value of slip, the shear stress is attained on the softening branch or on the residual stress branch. For all the analyzed cases, the stresses in the fiber are transferred to the matrix and entirely to the support.

**Figure 8:**
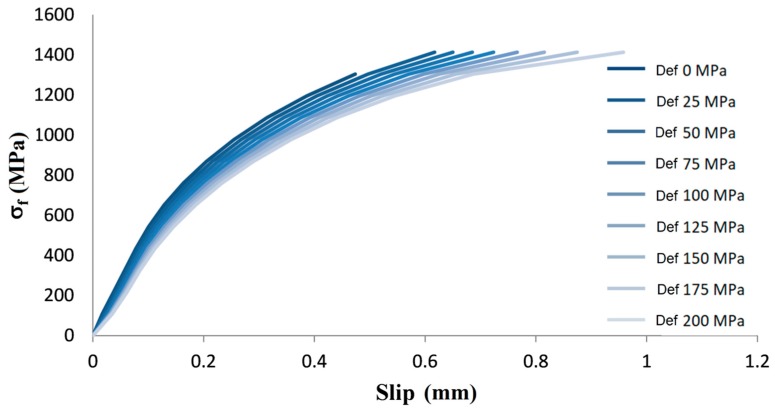
Stress-slip curves for different defect levels for PBO-FRCM.

The results of numerical simulation in Figure 8 allow to understand the typical variability of the experimental tests. The input properties are the same of the test setup of bond tests depicted in Figure 1 [16] (shear bond behavior of fibers into matrix can be further calibrated, but at date it is according to [27]) and the results show that bundles with defects reach the peak stress at different slip levels. When more bundles are stretched together, at each slip value, the average of the stress levels of bundles with different defects can be considered (i.e., parallel system). Obviously, if all the bundles have the same defects, the average curve corresponds to one of the curves in Figure 8, but if the realistic case of random defects is considered, the average curve is in between the curves and the peak can be attained at slip levels varying from about 0.4 to 0.8 mm).

## 4. Conclusions

The proposed approach is able to reproduce the variability typically found in the experimental bond tests. There is a bundle effect that justifies the scatter of the experimental data. The scatter found during experimental bond tests can be strictly related to the behavior of a single bundle immersed in a mortar prism analyzed in this work. Usually, groups of bundles are tested with a free portion pulled from outside the mortar matrix, being simultaneously pulled.

First of all, there are potential stretching defects even in the free portion, hence the bundles are not stressed at the same level, even if they are pulled together maintaining this initial stress offset, hence they could reach failure in a sequential order and not simultaneously.

Furthermore, stretching defect could also occur in the fibers embedded in the matrix and each bundle is characterized in a random way by its own application defect. As simulated in this work, by the proposed algorithm, the value of the slip where the fibers are embedded in the matrix is derived through the analysis of internal slips in the mortar. Therefore, the stress in each bundle during the bond test suffers at both stress scatters (in the embedded portion and in the free portion).

## Figures and Tables

**Table 1:** List of symbols—Geometrical and mechanical properties.

**Matrix (M)**	$A_{tm}$	Matrix Cross section effective in tension	mm^2^
	$t_{m}$	Matrix thickness	mm
	b	Matrix width/distance between bundles	mm
	$E_{m}$	Matrix Young’s modulus	MPa
	$f_{c}$	Compressive strength	MPa
	$f_{t}$	Tensile strength	MPa
	$\sigma_{m}$	Matrix stress	MPa
	$\varepsilon_{m}$	Axial strain in the matrix	-
**Fiber (F)**	Φ	Equivalent bundle diameter	mm
	$A_{f}$	Bundle Cross section	mm^2^
	$E_{f}$	Bundle Young’s modulus	MPa
	$\sigma_{f}$	Axial fiber stress	MPa
	$\varepsilon_{f}$	Axial fiber strain	-
	$\sigma_{f,def}$	Fiber defect stress	MPa
	$\varepsilon_{f,def}$	Fiber defect strain	-
**F-M interface**	$\tau_{RIG}$	Rigid shear stress	MPa
	$\tau_{MAX}$	Maximum shear stress	MPa
	$\tau_{RES}$	Residual shear stress	MPa
	$s_{1}$	Slip at $\tau_{RIG}$	mm
	$s_{2}$	Slip at $\tau_{MAX}$	mm
	$s_{3}$	Slip at $\tau_{RES}$	mm
**M-support interface**	$\tau_{MAX,SUPP}$	Maximum shear stress	MPa

**Table 2:** List of symbols—Parameter of the bond model.

*N*	Tensile force along the FRCM	N
*N*_0_	Tensile force in the FRCM at *x*_0_	N
Δ*x*	FRCM step discretization, hence distance between 2 control sections	mm
*s_n,TARGET_*	Slip target at the end of the FRCM, at *x_n_*	mm
*x_i_*	Current control section	-
*x_j_*	*x_i_* + Δ*x*	-
*u_f_*	Fiber axial displacement	mm
*u_m_*	Matrix axial displacement	mm
*s*	Slip between fiber and matrix	mm
*x*	Reference axis	-
*s_m,i−j_*	Average slip between fiber and matrix at *x_i_* and at *x_j_*	mm
*τ(s_m,i−j_)*	Average shear stress between fiber and matrix at *x_i_* and at *x_j_*	N/mm^2^
